# Pulmonary Hamartomas: A Single-Center Analysis of 59 Cases

**DOI:** 10.5152/eurasianjmed.2022.21150

**Published:** 2022-10-01

**Authors:** Ali Bilal Ulas, Yener Aydin, Atilla Eroglu

**Affiliations:** 1 Department of Thoracic Surgery, Atatürk University Faculty of Medicine, Erzurum, Turkey

**Keywords:** Lung, hamartoma, diagnosis, surgery

## Abstract

**Objective:**

This study investigated the clinical, radiological, and surgical treatment results of patients who underwent surgical treatment for pulmonary hamartomas.

**Materials and Methods:**

Fifty-nine consecutive patients who underwent surgical treatment for pulmonary hamartomas in our clinic between January 2001 and February 2021 were analyzed retrospectively.

**Results:**

Forty-three out of 59 (72.9%) of the cases were male and 16 (27.1%) were female. The average age was 52.0 ± 15.0 (between 5 years and 80 years). While pulmonary hamartoma was in the form of a solitary pulmonary nodule in 55 (93.2%) of the cases, there were multiple lesions in 4 (6.8%) cases. Simultaneous gastric adenocarcinoma was detected in 1 patient. One case had been operated on for Wilms tumor. Twenty-two (37.3%) of the cases were asymptomatic and were detected incidentally. Locations of pulmonary hamartomas were 18 (29.0%) in the left lower lobe, 16 (25.8%) in the right upper lobe, 12 (19.4%) in the right lower lobe, 9 (14.5%) in the left upper lobe, and 7 (11.3%) in the right middle lobe. The mean lesion diameter was 22.0 ± 9.5 mm (between 10 mm and 56 mm). Mild to moderate fluorodeoxyglucose (FDG) uptake was observed in 11 of 15 cases that were evaluated with positron emission tomography/computed tomography. Surgically, 44 (74.6%) patients underwent wedge resection, 13 (22.0%) patients underwent enucleation and two (3.4%) patients underwent lobectomy. Perioperative morbidity and mortality were not observed in any of the cases. The cases were followed up for an average of 40.6 ± 38.7 months (between 1 month and 151 months). No recurrence was observed in any of the cases during follow-up.

**Conclusion:**

Pulmonary hamartomas are usually detected incidentally and as a solitary pulmonary nodule. Although radiological findings provide important information, a definitive diagnosis is usually made during surgery. Parenchyma-sparing surgery should be preferred in these cases whenever possible.

**Main Points**

- Pulmonary hamartoma is a benign lung neoplasm that most commonly occurs in middle age or the elderly, with the highest incidence in the sixth or seventh decade of life.
- Most patients with pulmonary hamartoma are asymptomatic and detected incidentally on chest x-ray.
- It is estimated that the risk of lung cancer in patients with pulmonary hamartoma is 6.3 times higher than expected for the general population
- Surgery remains the only definitive treatment option today.

## Introduction

First described by the German pathologist Eugen Albrecht in 1904, hamartomas are benign tumors that can usually occur in the lungs, skin, heart, chest, and other parts of the body.^1^ The lesion contains an abnormal mixture of tissue components such as cartilage, epithelium, fat, or muscle.^1^ Hamartomas constitute 8% of solitary pulmonary nodules and 75% of benign nodules.^2^ It is usually detected incidentally on chest radiographs when cases refer to a physician for any reason. This study investigated the clinical, radiological, and surgical treatment results of patients who underwent surgical treatment for pulmonary hamartomas.

## Materials and Methods

Between January 2001 and February 2021, 59 patients underwent surgical treatment for pulmonary hamartoma in our clinic. Posteroanterior chest radiography and chest computed tomography (CT) was used for preoperative diagnosis in all cases. Additionally, positron emission tomography/computed tomography (PET-CT) was used in 15 (25.4%) cases, and magnetic resonance imaging (MRI) was performed in 12 (20.3%) cases. All cases had surgery under general anesthesia after double-lumen endotracheal intubation. The age, gender, comorbidity, location, number, size, radiological features of the hamartoma, surgical technique, morbidity, mortality, and long-term results of the patients were retrospectively reviewed.

Written informed consent was obtained from each patient. The study protocol was approved by the Atatürk University Faculty of Medicine Ethics Committee (B.30.2.ATA.0.01.00/426). The study was conducted under the principles of the Declaration of Helsinki.

### Statistical analysis

The IBM Statistical Package for the Social Sciences version 20.0 software (IBM Corp., Armonk, NY, USA) was used for statistical analyses. Data are presented as mean, standard deviation, number, and percentage.

## Results

Forty-three out of 59 (72.9%) of the cases were male and 16 (27.1%) were female. The average age was 52.0 ± 15.0 (between 5 years and 80 years). Only 1 (1.7%) of the cases was a child and the other 58 (98.3%) cases were adults. While pulmonary hamartoma was in the form of a solitary pulmonary nodule in 55 (93.2%) of the cases, there were multiple lesions in 4 (6.8%) cases (bilateral in 2 cases and multiple in the same lung in 2 cases). Simultaneous gastric adenocarcinoma was detected in 1 patient. One case had been operated on for Wilms tumor. Two patients had asthma and 1 patient had a history of tuberculosis. Twenty-two (37.3%) of the cases were asymptomatic and detected incidentally. Of the patients who presented with symptoms, 16 (27.1%) had shortness of breath, 14 (23.7%) had chest pain, 12 (20.3%) had a cough, 5 (8.5%) had back pain, and 5 (8.5%) had sputum.

**Figure 1.**
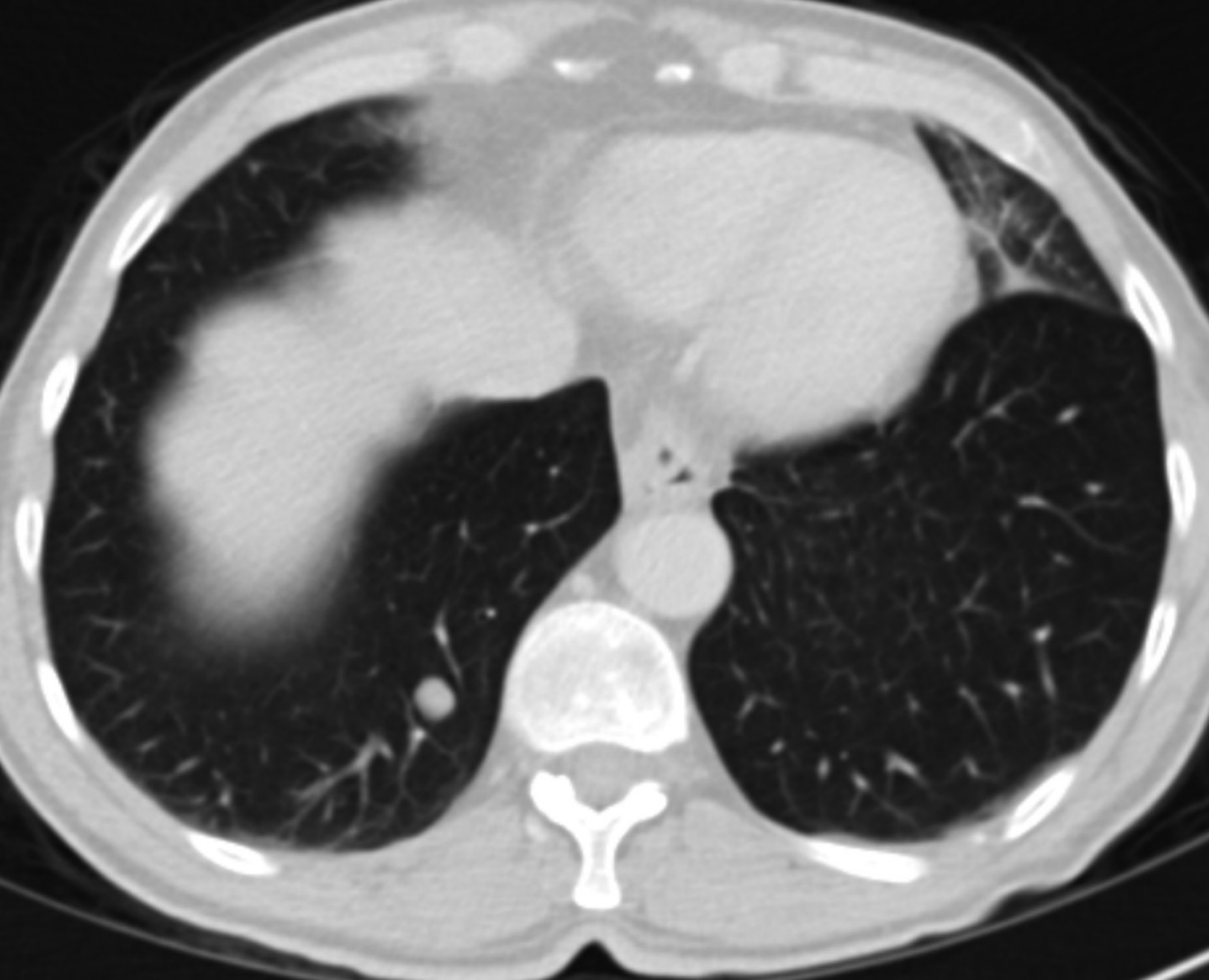
An axial chest CT section of a 57-year-old male patient shows an 11 × 10 mm nodule in the right lower lobe posterobasal segment. CT, computed tomography.

**Figure 2. a-c.**
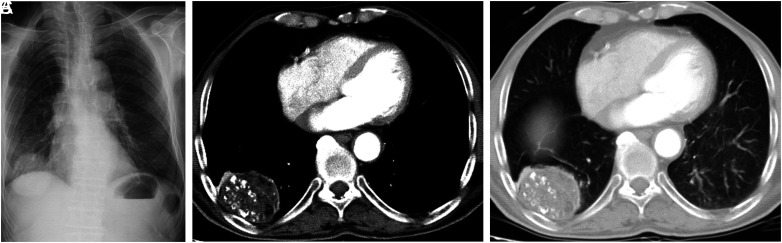
Chest x-ray radiography (a), chest CT scan in mediastinum window (b), and parenchymal window (c) sections show a solid mass with amorphous hyperdense calcified components in the right lower lobe superior segment in an 80-year-old male patient. CT, computed tomography.

Locations of pulmonary hamartomas were 18 (29.0%) in the left lower lobe, 16 (25.8%) in the right upper lobe, 12 (19.4%) in the right lower lobe, 9 (14.5%) in the left upper lobe, and 7 (11.3%) in the right middle lobe. Radiologically, 16 (27.1%) of the cases were calcified. While typical popcorn calcification was present in 6 (10.2%) cases, punctate calcification was detected in the remaining 10 (16.9%) cases. In 21 (35.6%) of the cases, an appearance in fat density was detected radiologically. The mean lesion diameter was 22.0 ± 9.5 mm (between 10 mm and 56 mm). While the diameter of the lesion was ≥30 mm in only 7 cases (11.9%), the diameter of the lesion was less than 30 mm in 52 (88.1%) cases (Figure 1 and 2). Mild to moderate FDG uptake was observed in 11 of 15 cases that were evaluated with PET-CT. Bronchoscopy was performed in 12 of the cases. No hamartoma was detected as endobronchial in any of the cases. A tru-cut biopsy was performed in 3 of the cases, but the histopathological diagnosis could not be obtained.

A total of 61 surgical procedures were performed in 59 cases (bilateral involvement in two cases). While 41 of the cases (67.2%) were approached by mini-thoracotomy, 20 cases (32.8%) were approached thoracoscopically. While 46 (75.4%) wedge resections were performed as intervention, enucleation was performed in 13 (21.3%) cases and lobectomy was performed in 2 (3.3%) cases. The mean operation time was 83.6 ± 25.9 minutes (between 40 and 165 minutes). Perioperative morbidity and mortality were not observed in any of the cases. The cases were followed up for an average of 40.6 ± 38.7 months (between 1 month and 151 months). No recurrence was observed in any of the cases during follow-up (Table 1).

## Discussion

Pulmonary hamartoma is a benign lung neoplasm that most commonly occurs in middle age or the elderly, with the highest incidence in the sixth or seventh decade of life. Generally, it is reported to occur 2-3 times more in men.^3-5^ Generally, the size of pulmonary hamartomas is less than 25 mm.^6,7^ In our series, there was only 1 child and all other cases were adults and the mean age was 52.0 years. The male : female ratio was 2 : 7. In accordance with the literature, the mean lesion diameter in our series was 22.0 mm.

Pulmonary hamartomas can be seen in the lung parenchyma and tracheobronchial tree. Endobronchial hamartomas account for only 1-19.5% of cases.^8-10^ In our study, hamartomas were localized in the parenchyma in all cases, and no endobronchial localization was observed.

Pulmonary hamartoma can occur in any lobe of the lung. It has been reported more particularly in the right lower lobe.^7^ In our series, they were seen in the left lower lobe in 29.0% of the cases, in the right upper lobe in 25.8%, and in the right lower lobe in 19.4%.

Most patients with pulmonary hamartoma are asymptomatic and detected incidentally on chest x-ray. It is difficult to determine whether the symptoms are related to the tumors. However, pulmonary hamartomas can cause atelectasis, infection, fever, and bleeding due to endobronchial compression or intraluminal enlargement. The main problem in asymptomatic patients is the difficulty in distinguishing pulmonary hamartoma from lung cancer.^10,11^ In our series, 37.3% of the cases were asymptomatic in terms of the respiratory system, 27.1% of the cases had dyspnea, 23.7% had chest pain, and 20.3% had a cough. However, these findings are thought to be due to other possible pulmonary causes and not hamartoma.

Pulmonary hamartoma usually occurs as a sharply circumscribed coin lesion with calcification on posteroanterior chest radiography. However, this is not diagnostic as calcifications can also be seen in carcinomas and tuberculosis.^12^ Despite the rapidly developing technology, it is often difficult to determine whether solitary pulmonary nodules are malignant or benign in high-risk groups on routine chest radiography or low-dose CT scans. Intranodular fat and popcorn-like chondroid calcifications are reliable indicators of hamartoma. However, fat is only detected in about 34% of hamartomas, and calcifications along with fat are present in about 21%.^2,7,13,14^ In general, various morphological features and their combinations may indicate a benign lesion on CT, but such features are not present in all benign nodules. Pulmonary hamartomas consist predominantly of cartilage; other components include fibromyxoid connective tissue, fat, bone, and smooth muscle. Esme et al^7^ reported that 37.5% of the cases had calcification and one-third of these calcifications had typical popcorn calcification and 29.1% of the cases had fat density. Calcification was observed in 27.1% of the cases in our series. A typical popcorn calcification rate was detected in 10.2% and fat density appearance in 35.6% of the cases.

De Cicco et al^2^ stated that, according to thoracic CT examination of patients with pathologically confirmed pulmonary hamartoma, 62% of the appearances were benign and 38% were malignant. In PET-CT scanning, they reported that 81% of the appearances were deemed benign, 9.5% suspicious and 9.5% malignant. They reported that PET-CT had uptake characteristics suggestive of malignancy in approximately 20% of cases, especially in large-sized nodules. Zhao and Wang^3^ compared PET / CT results in 105 patients with pulmonary hamartoma and 34 patients with carcinoid tumors. SUVmax value was found to be statistically higher in carcinoid tumors. However, in this study, the mean diameter of hamartomas was 16.2 mm, while the average diameter of carcinoid tumors was 23.8 mm. In our study, imaging was performed with PET/CT in 15 cases. Mild moderate FDG uptake was observed in 4 (26.7%) of the cases.

Flexible fiberoptic bronchoscopic transbronchial biopsy has also been proposed as a first-line technique in the diagnosis of pulmonary hamartoma. However, the usefulness of the recommendation for this procedure is controversial, as most hamartomas are located in the periphery of the lung and very few pulmonary hamartomas are endobronchial located.^15^ Bronchoscopy was performed on 12 cases in our study. However, a histopathological diagnosis could not be obtained because no endobronchial lesion was seen in any of them.

Multiple hamartomas are very rare, and there are studies reported as case reports in the literature.^16^ In our series, multiple hamartomas were detected in 6.8% of the cases.

Many studies are suggesting that there is an association between hamartoma and malignancy and the possibility that this may turn into carcinoma or sarcoma malignant. It is estimated that the risk of lung cancer in patients with pulmonary hamartoma is 6.3 times higher than expected for the general population.^4,7,8,17^ Also, synchronous or metachronous lung cancers may develop in patients with pulmonary hamartoma. Ekinci et al^4^ reported that they detected malignancy in 23 of 96 patients with pulmonary hamartoma. In our study, an association with malignancy was observed in 2 of 59 cases. Coexistence with lung cancer and subsequent development of lung cancer was not observed in any of the cases.

Today, despite the progress in medical treatment, pulmonary resection continues to be the most important treatment method in patients with hamartoma. However, there is still controversy about the indication and timing of the surgery. Because most hamartomas are non-growing or slow-growing neoplasms, some authors emphasize that surgery is only necessary for young or middle-aged patients when the lesion is enlarged or there are significant pulmonary symptoms. According to some authors, hamartomas tend to enlarge or recur and may contribute to the development of malignancy due to chronic inflammatory stimulation. Therefore, surgical resection is recommended when a solitary pulmonary lesion is greater than 25 mm or malignancy cannot be ruled out.^7,18,19^

Surgery remains the only definitive treatment option today. Preservation of functional lung tissue is the primary goal during surgery. Therefore, enucleation and wedge resection is the most commonly used surgical techniques. However, lobectomy and rarely pneumonectomy may be required in large, multiple, or centrally located lesions where wedge resection is not possible. In order not to miss the underlying malignant potential, an intraoperative frozen examination is generally recommended.^1^ Recurrences after hamartoma resection have been reported in the literature.^6^ In our study, while wedge resection was applied to 75.4% of the cases, enucleation was applied to 21.3% of the cases and lobectomy was performed in 3.3%. Pneumonectomy was not required in any of the cases. No recurrence was observed in any of the cases during follow-up.

One of the main limitations of this study is that it is a retrospective study. Another issue is the relatively small number of cases and the length of the study period.

As a result, pulmonary hamartoma is a benign and slow-growing tumor of the lung. They are usually detected incidentally and as a solitary pulmonary nodule. It may be difficult to radiologically distinguish tuberculosis from peripheral lung cancer. In most cases, the definitive diagnosis is made during the operation. Surgically, it should be approached as minimally invasive as possible and the parenchyma should be preserved. Enucleation and wedge resection are sufficient in most cases.

## Figures and Tables

**Table 1.** Patient Characteristics

	Number (n)	%
Gender		
Male	43	72.9
Female	16	27.1
Age (year)	52.0 ± 15.0	
Number of cysts		
Solitary	55	93.2
Multiple	4	6.8
Lesion size(mm)	22.0 ± 9.5	
Symptoms		
Dyspnea	16	27.1
Chest pain	14	23.7
Cough	12	20.3
Back pain	5	8.5
Sputum	5	8.5
Asymptomatic	22	37.3
Localization		
Left lower lobe	18	29.0
Right upper lobe	16	25.8
Right lower lobe	12	19.4
Left upper lobe	9	14.5
Right middle lobe	7	11.3
Radiological findings		
Popcorn calcification	6	10,2
Puncture calcification	10	16,9
Fat density	21	35,6
Surgical procedure		
Wedge resection	46	75.4
Enucleation	13	21.3
Lobectomy	2	3.3
Complications		
Yes	0	0
No	49	100
Mortality		
Yes	0	0
No	49	100
Recurrence		
Yes	0	0
No	49	100
